# The Vehicle Determines the Destination: The Significance of Seminal Plasma Factors for Male Fertility

**DOI:** 10.3390/ijms21228499

**Published:** 2020-11-12

**Authors:** Fengli Wang, Weina Yang, Sijin Ouyang, Shuiqiao Yuan

**Affiliations:** Institute Reproductive Health, Tongji Medical College, Huazhong University of Science and Technology, Wuhan 430030, China; wangfengli@hust.edu.cn (F.W.); yangweina@hust.edu.cn (W.Y.); ouyangsijin@hust.edu.cn (S.O.)

**Keywords:** seminal plasma, fertility, proteomics, microRNAs, immunomodulation, biomarker

## Abstract

Of all human infertility cases, up to 50% show contributing factors leading to defects in the male reproductive physiology. Seminal plasma (SP) is the biological fluid derived from the male accessory sex gland which carries spermatozoa passing throughout the male and female reproductive tract during ejaculation. It contains a complicated set of heterogeneous molecular structures, including proteins, cell-free nucleic acid (DNA, microRNA and LncRNA), and small-molecule metabolites as well as inorganic chemicals (ions). For a long time, the substantial significance of seminal plasma factors’ functions has been underestimated, which is restricted to spermatozoa transport and protection. Notably, significant advancements have been made in dissecting seminal plasma components, revealing new insights into multiple aspects of sperm function, as well as fertilization and pregnancy outcomes in recent years. In this review, we summarize the state-of-art discoveries regarding SP compositions and their implications in male fertility, particularly describing the novel understanding of seminal plasma components and related modifications using “omics” approaches and mainly focusing on proteome and RNA-seq data in the latest decade. Meanwhile, we highlighted the proposed mechanism of the regulation of SP molecules on immunomodulation in the female reproductive tract. Moreover, we also discussed the proteins investigated as non-invasive diagnosis biomarkers for male infertility in the clinic.

## 1. Introduction

Over the past few decades, worldwide, gradually decreased human fertility has attracted much more interest and became an important medical and social issue. Approximately 10–15% of couples of the reproductive-aged population are considered infertile, which is defined as the failure to conceive within at least one year of unprotected sexual intercourse [1,2]. For all infertility cases, up to 50% of them show contributing factors leading to defects in male reproductive physiology [3]. However, most male infertility causative factors remain elusive and the underlying mechanisms are also largely unknown. Thus, it is imperative to determine factors affecting male fertility, dissect regulatory mechanisms, search for useful diagnostic biomarkers, and, eventually, develop effective therapeutic tools.

To date, the majority of male infertility research has mainly focused on abnormality related to the testes, where the male gametes (spermatozoa) are produced through spermatogenesis and spermiogenesis [4]. For human male reproduction, apart from the testes, the accessory glands, including the prostate, seminal vesicles, and bulbourethral glands, are also essential, secreting a biological fluid termed as seminal plasma (SP) around sperm from ejaculation to the fertilization process [5]. Seminal plasma makes up more than 95% of human semen, whereas testicular secretions containing spermatozoa compose about 5%. Clinically, male fertility is often evaluated by routine semen analysis, which serves as a baseline marker with data regarding sperm quantity and quality, including sperm count, concentration, viability, motility, and morphology, while decreased fertility frequently associates with aberrant semen parameters [6].

**Figure 1:**
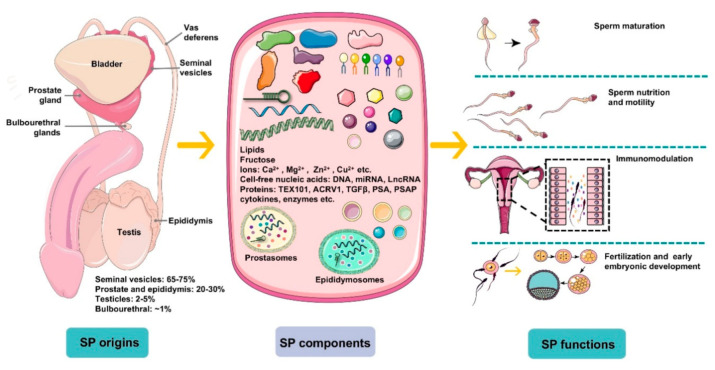
An overview of the origins, components, and main functions of seminal plasma (SP). Seminal plasma makes up more than 95% of the human ejaculate volume, whereas testicular secretions containing spermatozoa compose about 2–5% of semen. Seminal plasma is mainly derived from seminal vesicles, the prostate and epididymis, which produce ~65–75% and ~20–30% of the volume of semen, respectively, with a small proportion generated by bulbourethral glands (~1%). SP is composed of a complicated set of heterogeneous molecular structures, including proteins (enzymes, cytokines, TEX101, ACRV1, TGFΒβ, prostate-specific antigen (PSA), prostatic-specific acid phosphatase (PSAP), etc.), lipids, sugars (fructose), cell-free nucleic acid (DNA, microRNA, and LncRNA), ions (Ca^2+^, Mg^2+^, Zn^2+^, Cu^2+^, etc.), and small-molecule metabolites. In general, SP is a beneficial medium for spermatozoa maturation, nutrition, and motility and modulates spermatozoa function by regulating a cascade of molecular events, such as sperm maturation in the epididymis and capacitation during transport. More importantly, plasma molecules, such as cytokines, directly recognize receptors on epithelial cells lining the cervix and uterus to induce synthesis of pro-inflammatory cytokines and chemokines that recruit and activate inflammatory leukocytes. Besides, SP modulates the release of cytokines and growth factors that regulate embryo development in the oviduct and uterus before implantation, which is critical for early embryo development and implantation.

However, limitations exist in investigating male infertility exclusively using routine semen analysis parameters for a certain patient since unexplained male infertility (UMI) cases have an average prevalence of approximately 15% among all male infertility cases [7]. Unexplained male infertility refers to the diagnosis for an individual whose semen fulfills WHO criteria but fails to conceive offspring. Thus, it is necessary and urgent to develop novel evaluation tests assessing male factor infertility besides examining the structure and function of spermatozoa and, furthermore, the possible involvement of seminal plasma factors should be considered [8]. Seminal plasma is the fluid part of semen, which carries spermatozoa passing throughout the male and female reproductive tract during ejaculation, eventually reaching the oocyte for successful fertilization. It is composed of a complicated set of heterogeneous molecular structures, including proteins, lipids, sugars (fructose), cell-free nucleic acid (DNA, microRNA, and LncRNA), and small-molecule metabolites as well as inorganic chemicals (ions). In general, seminal plasma factors provide energy for spermatozoa metabolism and motility and modulate spermatozoa function by regulating a cascade of molecular events, such as sperm maturation in the epididymis and capacitation during transport. Plasma molecules can give an idea about sperm concentration, motility, morphology, and cause of infertility. Notably, emerging evidence has indicated that seminal plasma is not merely the beneficial medium of spermatozoa but also contains essential spermatozoa function modulators (Figure 1). Recently, advancing technologies in the “omics” fields, such as genomics, transcriptomics, proteomics, and metabolomics, have allowed uncovering of novel aspects and improved understanding of seminal plasma involved in male sub- and infertility.

This review provides an overview of the state-of-art discoveries regarding SP compositions and their implications in male fertility. We describe the novel understandings of seminal plasma components and related modifications using “omics” approaches, mainly focusing on proteome and RNA-seq data in the latest decade. We will also particularly highlight the functions of SP molecules for immunomodulation in the female reproductive tract and the proposed mechanisms. Subsequently, we discuss seminal plasma’s clinical relevance with particular reference to the potential application as a biomarker to better assess male fertility. Finally, the perspective of seminal plasma on male fertility is briefly summarized.

## 2. Protein Compositions of SP

Human normal seminal plasma is predominantly enriched with a large diversity of tissue-specific proteins and peptides, directly binding to spermatozoa, thus contributing to maturation, function, and interaction with the female genital tract [9,10]. Seminal plasma proteins are mainly derived from seminal vesicles and the prostate, which produce ~65–75% and ~20–30% of the volume of semen, respectively, with a small proportion generated by the testis, epididymis, and bulbourethral and periurethral glands [11]. The total protein concentration varies depending mainly on the detected technique at an average value of 40–60 mg/mL [12,13,14]. Prostasomes and epididymosomes are distinctive vesicles in SP secreted by the prostate and the epididymal epithelium, filled with a mixture of complex proteins. Prostasome proteome contains 139 proteins, revealed by microLC-MS/MS, including enzymes, transport/structural proteins, chaperone proteins, and signal transduction proteins [15]. The protein composition of epididymosomes differs from prostasomes, which contain 146 different proteins identified by MS [16]. High-abundance proteins in human seminal plasma, such as matrix and adhesion proteins semenogelins, laminin, and enzymes, including prostate-specific antigen (PSA), prostatic-specific acid phosphatase (PSAP), and creatine kinase, can be identified by 2D gel electrophoresis or mass spectrometry (MS) [17,18]. The application of advanced proteomics technologies has gained substantial progress to elucidate relative or absolute quantitation of seminal plasma protein composition and content and provided the underlying mechanistic insights of male reproductive physiology and pathology [7] (Table 1). The proteomics approach largely supports the discriminate differences in protein composition of semen, based on which proteins can potentially be used for noninvasive clinical diagnosis for male infertility disorder.

SP protein profiling is dynamic and affected by various factors, such as reactive oxygen species (ROS). Aberrant ROS level is a common consequence of diverse pathways that cause sperm dysfunction in the semen of two-thirds of infertile patients [40,41]. One-dimensional gel electrophoresis-coupled LC/MS–MS identified 105 unique, differentially expressed proteins from high-ROS infertile patients compared with fertile controls, which is much more than a comparison between medium-ROS or low-ROS patients with a control [42]. Rakesh Sharmae et al. categorized seminal plasma into ROS-positive (ROS+) or ROS-negative (ROS−) groups from both fertile and infertile men and then profiled proteins to uncover molecular mechanisms underlying oxidative stress and sperm dysfunction. They identified four ROS+−specific proteins: cystatin S precursor, albumin preprotein, lactotransferrin precursor-1 peptide, and prostate-specific antigen isoform 4 preprotein [35].

Besides, distinct protein content in SP is associated with semen parameters. Many studies have explored the association of seminal plasma protein profile and routine semen analysis. Analysis of the proteins profiled in the following groups: (1) NN (normal sperm count and normal morphology), (2) NA (normal sperm count and abnormal morphology), (3) ON (oligozoospermia and normal morphology), and (4) OA (oligozoospermia and abnormal morphology) by LC-MS/MS revealed 20 proteins differentially expressed, which indicates a correlation between seminal plasma protein and sperm concentration and morphology [43]. It is reasonable that SP protein is associated with sperm motility because sperm are surrounded by SP and directly bound by specific proteins on the surface. From asthenozoospermic patients, a total of 29 differentially expressed proteins were identified by quantitative proteomes, which could be used as candidate targets for studying the molecular bases of sperm motility [20].

Proteins in seminal plasma are closed related to male fertility status and, thus, may provide predicted information of male reproductive health. Investigating the differential expression of proteins of distinct fertility states can provide potential mechanism implications underlying the decreased male fertility. In oligoasthenozoospermic patients, a proteome comprising 734 proteins in seminal plasma was established with 22 upregulated, and 20 downregulated through comparative proteomics using the label-free high-throughput iTRAQ approach [27]. Another study on seminal extracellular vesicles proteome from asthenozoospermia patients identified a list of 91 differentially expressed proteins, of which 11 proteins were significantly upregulated and 80 were downregulated [23]. For a male with primary infertility and one with secondary infertility, 48 and 53 proteins in seminal plasma were differentially expressed to the control group, respectively. ANXA2, CDC42, and SEMG2 are dysregulated in primary infertility and ANXA2 and APP were overexpressed in secondary infertility, which may serve as diagnostic biomarkers [44].

An abnormal male reproductive system anatomy structure induces the alteration of SP proteins. Proteomic analysis demonstrates that 91 proteins are differentially expressed in varicocele compared to 12 months after varicocelectomy samples from the same individuals [45]. In prostatitis patient seminal plasma, a total of 59 proteins were identified, 33 of which were significantly increased, whereas 26 were decreased in prostatitis compared to normal controls [46]. In varicocele-associated infertility, such as bilateral varicocele patients, global proteomic analysis of SP proteins revealed that the SP homeostasis is compromised due to the dysregulation of proteins involved in ROS response and tissue homeostasis compared with healthy men [19]. Further, it seems that smoking has a synergistic effect with varicocele on SP proteome, and a cross-sectional study revealed that varicocele smokers presented lower mitochondrial activity and acrosome integrity and higher DNA fragmentation compared with non-smokers. Ninety-one proteins, including neprilysin, beta-defensin 106A, and histone H4A, were dysregulated, which indicated that smoking triggers the establishment of inflammatory protein pathways in the testis/epididymis with varicocele [47]. Additionally, for men with normal fertility, smoking has an adverse impact on functional aspects of sperms such as sperm DNA damage and the destruction of acrosomes’ integrity. Proteomic analysis by LC/MS detected 422 proteins, of which 34 are differentially expressed and associated with an inflammatory state [31]. Obesity is another factor resulting from an unhealthy lifestyle which is believed to be potentially related to male fertility decline. Proteomics analysis of seminal plasma from obese men revealed 69 proteins differentially expressed, enriched in pathways such as apoptosis, activation of immune and inflammatory responses, and antioxidant activity compared with males with a normal BMI. Simultaneously, sperm in the obesity group presented decreased non-progressive motility and acrosome integrity and increased sperm DNA fragmentation [25].

In addition to protein expression, post-translational modification is a remarkable aspect of protein function regulation. Glycoproteins on the cell surface predominantly mediate cell–cell and cell–protein interactions. A typical example is Gd-S, the male-specific form of glycodelin S (Gd-S), highly expressed in seminal vesicles [48]. Glycosylated glycodelin binds to the sperm to inhibit capacitated until ejaculation pass through the cervix. Conversely, reversible binding enables the immediate disassociation of Gd-S from the sperm surface immediately when capacitation and fertilization are demanded [49]. Employing enrichment of N-linked glycosylated peptide-coupled LC-MS/MS analysis, Yang et al. found the first N-linked glycoproteome in human SP, in which 372 proteins were identified at 720 N-glycosylated sites [32].

## 3. Cell-Free Nucleic Acids in SP

Seminal plasma cell-free nucleic acids mainly include cell-free DNAs (cfDNAs) and cell-free RNAs (cfRNAs), comprising messenger RNAs (mRNAs) and long non-coding RNA (LncRNAs), microRNAs (miRNAs), and piwi-interacting RNAs (piRNAs). cfDNA can be detected in semen by modified capillary gel electrophoresis (CE) followed by SYBR-Gold staining. Interestingly, low-molecular-weight seminal cfDNA content is significantly positively associated with sperm rapid progression, curvilinear velocity, morphology, and capacitation index [50]. The average concentration of cfDNA from normozoospermic semen is 1.34 ± 0.65 μg/mL, whereas a higher concentration can be detected in azoospermia with a value of 2.56 ± 1.43 μg/mL [51]. Moreover, testis-specific methylated gene promoters (*CCNA1*, *ACRBP*, CIB1, *DMRT1*, and *HSF1*) can be detected from seminal cfDNA, which correlated with methylation percentage in testicular DNA and displayed dynamic changes among nonobstructive azoospermia (NOA) patients and normal controls [52]. This study demonstrated that cfDNA carries epigenetic information originating from the testes and could be a candidate as a non-invasive biomarker for spermatogenesis abnormalities. More recently, a mitochondrial DNA copy number in seminal plasma was detected in semen, significantly decreased in asthenozoospermia and oligoasthenozoospermia patients compared with normal controls, and positively correlated with semen parameters, such as sperm concentration and motility [53].

MicroRNAs (miRNAs) are a type of small-molecule RNA that are widely involved in a variety of physiological pathways of the body, including stress response, cell differentiation, cell metabolism, cell apoptosis, and the generation and metastasis of tumors [54] as well as the much-implicated spermatogenesis process [55,56]. Recent growing studies have uncovered abundant and stable miRNAs in seminal plasma, but only about 20% of free miRNAs are stored in microvesicles (MV) and exist in the form of seminal corpuscles, and most of the remaining seminal plasma is free miRNA that exists as a protein complex [57]. Simultaneously, the seminal plasma miRNA expression profile of male infertile patients is significantly different from normal controls. Some specific seminal plasma miRNA changes are closely related to male infertility and spermatogenic disorders, which can be used as new diagnostic markers of male infertility. The study of seminal plasma miRNA of specific changes can provide a new direction for studying the molecular mechanism of male infertility (Table 2).

In 2011, 19 miRNAs with a significant difference in seminal plasma between infertile and fertile men were screened by Wang et al., of which seven kinds of miRNA (miR-34c-5p, miR-122, miR-146b-5p, miR-181a, miR-374b, miR-509-5p, and miR-513a-5p) were significantly decreased in azoospermia patients [68]. Subsequently, in another study, Wu et al. found that the expression of miR-19b and miR-7a was significantly upregulated in the seminal plasma of NOA patients, but there was no significant difference between asthenospermia patients and normal controls [69]. Further, in vitro experiments showed that miR-141 down-regulated the expression of CBL and TGFβ-2 protein, and miR-7-1-3p down-regulated the expression of RB1 and PIK3R3 [70]. It has been reported that there are significant changes in miRNA in the epididymis and seminal plasma of vasectomized men [71]. The above results show that the seminal plasma miRNA expression profile of male infertile patients is significantly different from that of the normal control group and the seminal plasma miRNA with specific changes can be used as a potential marker for the diagnosis of male infertility.

For patients with sperm disorders caused by varicocele, miR-210-3p can be used as a biomarker for screening, which is the key to determine effective early treatment and protect patients’ fertility [62]. miR-192a induces apoptosis of GC-2 cells by activating caspase-3 protein, which is also a potential marker to successfully indicate the sperm present in patients’ ejaculatory fluid with varicocele after microsurgery [64]. Indeed, the levels of miR-122, miR-181a, and miR-34c-5p in seminal plasma were significantly decreased in infertile men with varicocele and oligozoospermia and were related to apoptosis markers (BAX and BCL2) and oxidative stress (OS) [72].

Since the content of RNA molecules, especially miRNA, in the seminal plasma of exosomes changes with the origin cell, it can accurately reflect the pathological and physiological changes of reproductive organs and can be used as a potential reliable biomarker. Using ultracentrifugation technology, Abu-Halima et al. isolated extracellular microbubbles, including exosomes from seminal plasma, analyzed the expression of 1205 miRNAs by chip technology, and identified 36 miRNAs with altered expression levels, including 7 miRNAs with high expression and 29 with low expression in oligoasthenospermia [73]. Recently, Barcelo et al. compared the miRNA expression profiles of seminal plasma exosomes between patients with different pathological types of azoospermia (obstructive azoospermia and endocrine azoospermia). Five miRNAs (miR-182-3p, miR-205-5p, miR-31-5p, miR-539-5p, and miR-941) were found to be potential biomarkers in patients with endocrine azoospermia [74]. The above results suggest that the change in miRNA expression in seminal plasma exosomes will contribute to the development of male infertility molecular markers and molecular mechanisms.

The sperm DNA fragmentation index (SDF), as an important supplement to semen routine parameters, has been proposed to distinguish fertile and infertile men and to predict the outcome of natural pregnancy and in vitro fertilization. Che et al. found that the level of miR-424 (mouse homologous miR-322) in the seminal plasma of infertile patients with high DNA fragmentation index (DFI) was much lower than that of the fertility group and established a GC-2 cell model of down-regulation of miR-322, which leads to spermatogenesis disturbance. It was observed that after inhibition of miR-322, the survival rate of GC-2 cells decreased significantly and apoptosis increased significantly. MiR-322 plays a crucial role in promoting the apoptosis of GC-2 cells by directly regulating the expression of *DDX3X*. The downregulation of miR-424 expression in infertile men may directly affect spermatogenic cell apoptosis and sperm DNA damage [60]. Other studies have shown that the decreased expression of miR-374b and miR-26b can be used as the first indicator of the increase in SDF and they can be used as auxiliary biomarkers to diagnose male idiopathic infertility [75].

Long non-coding RNAs (lncRNAs) are a class of RNAs with a length longer than 200 nucleotides, acting as gene expression regulators from both transcription and post-transcription aspects [76,77]. RNA-sequencing of seminal plasma exosomes revealed the existence of lncRNAs, some of which differentially expressed in asthenozoospermia compared with a normal group, indicating a pivotal role of lncRNAs as molecular mechanisms of asthenozoospermia [78]. In addition, the expression profile of extracellular vesicle lncRNAs in nonobstructive azoospermia (NOA) patients is significantly altered, of which a set of differentially expressed testis-specific lncRNAs displayed valuable prediction potential to assess the presence of testicular spermatozoa in NOA patients [79].

## 4. Regulation of Hormones on SP Components

Sex steroid hormones, including androgen and estrogen, are present in seminal plasma, in cooperation with other constituents necessary for normal spermatogenesis, protection, and spermatozoa maturation. As early as the 1970s, levels of steroid hormones in seminal plasma have been determined by many studies [80,81]. As for testosterone, the range of its concentration in seminal plasma from normal persons is very broad due to the methodology employed rather than to differences in sperm parameters [81]. In most of the earlier papers, no significant testosterone content differences were observed between normal and impaired spermatogenesis [82,83,84,85]. However, later refined studies discriminated the correlation between sperm abnormalities (oligo-, astheno-, and azoospermia) and hormone concentrations. Testosterone levels in infertile patients (oligozoospermia, obstructive azoospermia (OA), and nonobstructive azoospermia (NOA)) are significantly lower than those of normospermia men [86]. For varicocele, it is still controversial whether seminal testosterone concentration is altered or not. One study stated an observed slightly higher testosterone concentration in men with varicocele [87], but another reported no significant differences in varicocele patients from controls [88].

Alterations in SP hormone profiles are associated with elevated sperm abnormalities. Seminal testosterone and dihydrotestosterone concentrations were significantly lower in patients with abnormal sperm characteristics, such as sperm concentration and motility, than in men with normozoospermia [89]. The effect of hormone changes on other compositions in seminal plasma has been explored. In secondary male hypogonadism patients with significantly lower testosterone, estradiol (E2), luteinizing hormone (LH), and follicle-stimulating hormone (FSH) in serum, 33 proteins were missing among the 61 proteins detected in the normogonadal men, which indicated the effect of hormones on the secretory function of male accessory glands. Further, 14 proteins reappeared after 6 months of testosterone replacement therapy (TRT), confirming testosterone regulation on seminal plasma constituents [90]. Another study on seminal plasma protein profiling of hypogonadotropic hypogonadism patients showed 11 proteins’ levels were decreased compared with controls, which were mainly involved in hydrolase activity and protein-binding activity, whereas the levels of six proteins were recovered after testosterone replacement therapy (TRT) [21]. Notably, the significantly reduced levels of prostatic-acid phosphatase, zinc, prostate-specific antigen, lactoferrin, and fructose in seminal plasma were observed after testosterone administration [91]. In addition, a single injection of testosterone enanthate (TE) in oligospermic men led to increased ornithine decarboxylase activity and higher levels of fructose and sialic acid [92].

Apart from testosterone, other hormones, such as estradiol, human chorionic gonadotropin, Anti-Müllerian hormone (AMH), and relaxin, also are presented in seminal plasma. For infertile men, the estradiol level was significantly increased [85]. Specifically, estradiol levels in OA patients are significantly higher than those in normospermic and NOA cases [86]. AMH is a glycoprotein produced by Sertoli cells that belongs to the transforming growth factor (TGF) beta superfamily. Thus, it is reasonable that seminal AMH is undetectable in all OA patients due to its origin, and besides, in NOA patients, seminal AMH concentration was also significantly lower and may serve as a seminal marker for spermatogenesis [93]. In addition, total AMH (pmol/ejaculate) content in seminal plasma was positively correlated with sperm concentration and sperm count [94,95]. For hCG, its free beta-subunit levels are significantly lower in infertile patients than those in controls and correlated with sperm count and sperm motility [96]. Relaxin is a hormone that contributes to pregnancy and parturition in females and can be detected in seminal plasma at a concentration of approximately 50 ng/mL, which is mainly produced by the prostate gland [97,98]. Relaxin can positively affect sperm functions related to fertilization ability [99]. However, there are disagreements regarding relaxin’s effects on sperm motility. Some groups indeed observed statistically significant effects of relaxin on human sperm motility [100,101], but other studies did not present effective data of relaxin’s effect on the motility of sperm [102,103].

## 5. SP Factors and Related Pathways

The interactions among SP factors composing signaling pathways make their functions more complicated; thus, it is challenging to dissect each factor and related pathways in the modulation of sperm function and interaction with the maternal reproductive tract. Several signaling pathways have been reported to implicate seminal plasma factors. For example, extracellular Zn^2+^ in seminal plasma binds to G protein-coupled receptor 39 (GPR39)-type Zn-receptor and activates the AC-cAMP-PKA-Src-EGFR signaling cascade, which is essential for hyperactivated motility (HAM) during sperm capacitation [104]. Besides, Fms-like tyrosine kinase 3 (FLT3) is a type III kinase enriched in seminal plasma and highly expressed in infertile men. Its inhibition can repress fertilization and early embryonic development through a PKA-dependent pathway, which indicates that FLT3 is a key factor for male fertility [105]. Alterations in distinct pathways can be induced by internal healthy state and external factors. Obesity negatively regulates proteins enriched in the intrinsic pathway of apoptosis, activation of immune and inflammatory responses, and antioxidant activity [25]. In bilateral varicocele patients, compared with healthy men, pathways involved in response to reactive oxygen species and oxidative stress and tissue homeostasis were affected [19]. Smoking influences inflammatory state-linked signaling pathways, such as antigen processing and presentation, protein kinase A signaling, complement activation, the cytokine-mediated signaling pathway regulation, and acute inflammatory response regulation, which indicates the inflammatory activity of seminal plasma in coping with cigarette toxicity [31].

## 6. SP Factors Function as Immunomodulators in the Maternal Reproductive Tract

It is well known that sperm is an auto-antigen and an alloantigen in both men and women, and immune factors also play an important role in infertility. However, due to a series of immune protection mechanisms, including a series of immunological mediators found in seminal plasma in recent years, this avoids the occurrence of the autoimmune phenomenon and fertilized egg rejection by affecting maternal immune cell function [106]. Cellular integration between the immune system and the reproductive system is a basis for normal male reproductive physiology. Cytokines are part of the male reproductive tract’s autocrine/paracrine network, which plays a vital role in testicular function and spermatogenesis. IL-6 is the core member of cytokines, which is produced by activated T lymphocytes, peripheral blood monocytes, macrophages, vascular endothelial cells, and smooth muscle cells, and participates in inflammation and immunity. Seshardi et al. and Camejo et al. reported an increase in pro-inflammatory IL-6 levels in SP in infertile patients [107,108]. Interestingly, Seshadri et al. found that the levels of IL-6 in mild and severe oligozoospermia, and IL-8 and IL-10 levels in asthenospermia, were significantly increased. In addition, Qian et al. found that the level of IL-11 in the semen of the infertility group, which was positively correlated with sperm viability, motility, survival rate, and normal sperm morphology but negatively correlated with IL-17 and IL-18 levels, was significantly lower than that of the normal sperm group [109].

**Figure 2:**
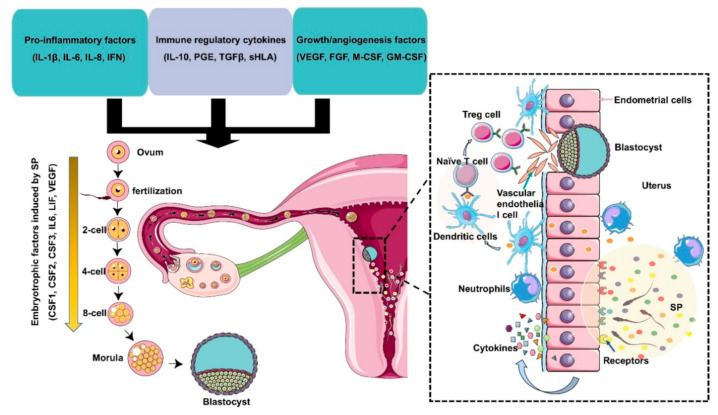
Immunomodulation of SP factors in the maternal reproductive tract to facilitate early embryo development and implantation. Seminal plasma contains chemokines, cytokines, and prostaglandins produced by Leydig and Sertoli cells, seminal vesicles, leukocytes, and other immune cells present in the male reproductive tract. These factors potentially induce inflammation, immunotolerance, and angiogenesis to support embryo development and implantation through the upregulation of embryokines, chemokines, and cytokines from oviductal and uterine tissues. Binding of active moieties in SP to endothelial cells stimulates the recruitment of neutrophils into the uterine tissues, which can clear up excess sperm and seminal debris. Moreover, dendritic cells can recognize paternal major histocompatibility antigens and pass them onto naive T cells and generate an expanded T regulatory (Treg) cell pool in lymph nodes. Since the embryo presents the same paternal antigens in the SP, the response of Tregs to these antigens can be efficiently suppressed, which allows implantation of the semiallogenic embryo into the maternal uterine. On the other hand, Tregs also contribute to endometrial tissue remodeling and receptivity for implantation through the regulation of angiogenesis. SP also results in the production of cytokines and growth factors, such as colony-stimulating factors (CSFs), leukemia inhibitory factor (LIF), and interleukin 6 (IL-6), by the maternal reproductive tract, which are essential for promoting embryo development before implantation. The arrow indicates that embryotrophic factors (CSF1, CSF2, CSF3, IL6, VEGF) are induced by SP during the early embryonic development.

Transforming growth factor-β is produced by seminal vesicles and the prostate and its content in seminal plasma is several times higher than in serum. The TGF-β family includes three polypeptides, TGF-β1, TGF-β2, and TGF-β3, all of which exist in human SP. TGF-β1 is the most abundant TGF-β [110]. It is reported that the levels of TGF-β1 and IL-18 in seminal plasma are related to the reproductive outcome of patients exposed to SP during in vitro fertilization (IVF) and intracytoplasmic sperm injection (ICSI) [111]. It can be seen that cytokines rarely play a role alone, rather they play a role in a complex network, directly or indirectly affecting sperm function. The relationship between cytokines and the reproductive system is mutual. All kinds of cells in the reproductive system can produce cytokines on their own and regulate the secretion of cytokines. If the secretion of cytokines is abnormal, it may lead to the damage of reproductive function, which may be due to the increase or decrease in suppressor T lymphocytes’ activity and the activation of helper T cells. At present, many studies have confirmed that seminal plasma immune factors are closely related to infertility and reproductive outcome. Future monitoring of more cytokines and other immune factors in seminal plasma will provide evidence for a better understanding of the mechanism of infertility (Figure 2).

Under the factors of injury, infection, and obstruction, the abnormal opening and regulation of the blood–testis barrier lead to the contact of spermatocytes, spermatids, and sperm with the immune system of the body, resulting in autoimmune response and the formation of various types of anti-sperm antibodies (ASA). Sperm autoimmune reaction, which produces anti-sperm antibodies, is also one of the causes of male infertility, accounting for about 10–30% of infertile patients [112,113]. In normal spermatozoa, the sperm autoimmune response leads to a decline in fertility caused by the acrosome reaction (AR) and dysfunction of capacitation and DNA fragmentation. The damage of sperm quality in immune infertility is more obvious: decreased sperm concentration and motility, morphological changes, acrosome reaction damage, and DNA fragmentation. The possible pathogenesis of these damages is related to oxidative stress (OS) [114]. Researchers have found that the production of AsAb is closely related to the level of the cytokine TNF-α in infertile patients, and the levels of AsAb and TNF-α in seminal plasma are significantly higher than those in serum, which indicates that local immune activation and immune damage in reproductive tract play a vital role in the occurrence of male infertility [115]. ASA is also found in the seminal plasma of patients with varicocele. In evaluating fertility recovery after varicocelectomy, anti-sperm immune response reduces the effect of varicocelectomy on reproductive function recovery; the higher the proportion of ASA, the lower the grade of varicocele and the worse the prognosis [116]. A systematic review and meta-analysis showed that sperm anti-sperm antibody was not associated with pregnancy rate after IVF or ICSI, indicating that these two kinds of assisted reproductive technologies are still feasible options for infertile couples with anti-sperm antibodies [117].

There is an immunosuppressive substance in seminal plasma, seminal plasma immunosuppressive factor (SPIF), which can block and change sperm membrane antigen and inhibit the immune system, such as lymphocytes, NK cells, macrophages, and complement systems [118,119,120]. SPIF is one of the crucial factors to protect sperm. Under normal circumstances, SPIF can protect sperm from the attack of immune cells and autoimmune responses and play an immunological role in the male reproductive system, ensuring the reproductive process’ smooth progress. The lack of immunosuppressive factors in male sperm can lead to the production of anti-sperm antibodies, resulting in infertility, or the spouse develops an allergic reaction or anti-sperm antibody after sexual intercourse [121,122,123]. At the same time, the existence of SPIF also promotes the occurrence, infection, and transmission of some diseases (such as AIDS). When the activity of SPIF is too high, the recognition and killing effect of immune factors on malignant tumors and pathogenic microorganisms is weakened and it is easy to spread sexually transmitted diseases (STDs); if the activity of SPIF is too low, it cannot effectively inhibit the killing effect of immune factors on sperm, resulting in the decrease in sperm motility and viability and the implantation ability of fertilized eggs. In some conditions, such as reproductive tract infection or trauma, SPIF can induce the body to produce anti-SPIF antibodies so that the amount of SPIF is relatively reduced, resulting in low SPIF activity. Vanage et al. found that SPIF antibody could induce sperm agglutination mediated by antibody, resulting in decreased fertility, and determined the positive rates of anti-SPIF-IgG and IgA; anti-SPIF-IgG levels in the seminal plasma of infertile men were slightly higher than those of IgA but there was no significant difference. This study suggested that there may be two sources of anti-SPIF antibodies in seminal plasma [120].

## 7. Potential Application of Seminal Plasma Factors as Bio-Markers

It is promising that seminal plasma proteins might be sources of biomarkers for the noninvasive diagnosis of male fertility disorders due to their relatively high levels and ease of collection. Aberrant protein concentration changes in seminal plasma can indicate pathological process stages and discriminate different types of male infertility. The verified and validated protein biomarkers are L-PGDS, ACRV1, ECM1, and TEX10. L-PGDS is a 26KDa enzyme and glycoprotein, mainly secreted by Sertoli cells, with unclear male fertility function. ELISA determined the seminal concentration of this enzyme ranging from 0.3 to 42 μg/mL, which was significantly lower in the oligozoospermic group (2.47 ± 0.51 μg/mL) than in the normozoospermic group (9.75 ± 1.49 μg/mL) [124]. A more detailed association study revealed that the concentration of seminal L-PGDS is significantly positively correlated with sperm concentration, sperm motility, and percentage of normal morphology and decreased progressive motility from normal to oligospermic patients [125]. Male infertility can be classified into oligozoospermia, asthenospermia, and azoospermia, based on the number and motility of sperm, among which azoospermia is the most severe of male infertility, with near absence of sperm in semen. SP-10 is an acrosomal matrix protein encoded by the acrosomal vesicle protein 1 (*ACRV1*) gene, which is specifically expressed in spermatids after meiosis and sperm [126]. ELISA using monoclonal antibody revealed a direct relationship between sperm count and seminal concentration of SP-10, which permits it to be a biomarker for oligozoospermic diagnosis. SpermCheck Fertility, a reliable and straightforward immunodiagnostic test based on the above idea, was developed, which quickly provides information for males on whether their sperm number is normal or not to evaluate their fertility [127], and the clinical and consumer trial of SpermCheck Fertility has been applied to detect extreme oligospermia or azoospermia [128].

To date, testicular biopsy is the only valuable method to detect whether spermatozoa are in the testis or not and to distinguish obstructive azoospermia (OA) and nonobstructive azoospermia (NOA). However, surgical exploration of random testicular tissue may not accurately reflect NOA histopathology because of the spatial distribution of spermatogenesis [129]. Searching for biomarkers may allow for avoiding the painful biopsy and deviation of the diagnosis results. Fortunately, some proteins can potentially differentiate normal, OA, and NOA patients. Seminal L-PGDS level could be applied as a biomarker for azoospermia, and high L-PGDS (more than 100 g/L) in men with azoospermia could be potentially diagnosed as nonobstructive azoospermia without biopsy, which contributes to infertility in almost 30% of these men [130]. The concentrations of epididymis-specific expressed ECM1 in OA patients are significantly lower than in control and NOA cases and, more importantly, could distinguish OA cases from normal ones with 100% specificity and sensitivity, and OAs from NOAs with 73% specificity, at 100% sensitivity at a cutoff of 2.3 mg/mL [129].

TEX101 is a membrane glycoprotein and is specifically expressed in germ cells without any expression in other tissues and cells, which can be cleaved from the spermatozoa surface and released into seminal plasma during sperm maturation into epididymis [131]. The notably decreased concentration in azoospermia makes it valuable as a biomarker to discriminate azoospermia from normal controls. Furthermore, the specific expression pattern allows it to differentiate various histopathological NOA subtypes, including hypospermatogenesis (HS), maturation arrest (MA), and Sertoli cell-only syndrome (SCO). In fact, the average concentration of seminal TEX101 from normal men is approximately 2 mg/mL, whereas there are low levels in SP (<120 ng/mL) in NOA cases with HS and MA, and it is not detectable in SCO without germ cells [129].

## 8. Conclusions and Perspective

To summarize, this paper reviewed the latest achievements of proteins and cell-free nucleic acids present in seminal plasma and the immunomodulation function in male infertility. Standard semen parameters are poor predictors of reproductive outcomes. Clinically employing the “gold-standard” semen analysis provides limited sperm information and cannot discriminate fertile from infertile men because of individual bias. Seminal factors contributing to men’s infertility have been explored over the past few decades, along with developing tools to evaluate and diagnose infertility. Although the emerging “omics” technologies promised to validate or discover known or novel factors, due to the complexity and variability of seminal plasma composition, it is still challenging to determine their origins from accessory sex glands and the proper functions contributing to sperm and to explain the underlying molecular mechanisms. Encouraging signs of progress have been made in the development of biomarkers in seminal plasma, for instance, the success of commercial TEX101- and ECM1-based immunodiagnostic assays with high specificity and sensitivity for azoospermia. Researchers will keep working on exploring and validating the function of SP factors to develop biomarkers for male infertility diagnosis and clinical consultation.

## Figures and Tables

**Table 1:** Distinct proteomes of human seminal plasma in respective conditions reported from 2012 to 2019.

Year	Sample Types	Proteomics Technique	Outcome or Significance	Reference
2019	Bilateral varicocele	LC-MS/MS	Identified altered DEPs and proposed potential noninvasive markers for varicocele patients.	[19]
2019	Normospermic Asthenozoospermic	LC-MS/MS	First investigation of the absolute expression features of low expressed proteins and identified 29 differentially expressed proteins related to sperm motility.	[20]
2019	Normal fertility Hypogonadism	HPLC/MS	Identified proteins that might represent putative clinical markers for patients with distinctive grades of male hypogonadism.	[21]
2019	Unexplained male infertility (UMI)	2D-PAGE/ MS	Altered levels of clusterin, epididymal secretory protein E1, and prostate-specific antigen were observed and might be introduced as new candidate biomarkers for success of IVF in UMI couples.	[22]
2019	Normospermic Asthenozoospermic	iTRAQ	Identified 3699 proteins in the seminal extracellular vesicles, and TRPV6 was markedly reduced in asthenozoospermic patients.	[23]
2019	Unilateral Varicocele	1D-PAGE/ LC-MS/MS	First report to identify DEPs in seminal plasma of unilateral varicocele patients, and uncovered KIF5B and ANXA2 potentially as biomarkers of infertility in unilateral varicocele.	[24]
2019	Non-obesity Obesity	LC-MS/MS	Obesity caused 69 differentially expressed proteins compared with controls.	[25]
2018	*Enterococcus faecalis* (*E. faecalis*) infection	HPLC/MS	First application of MS-based proteomics to uncover proteins reflecting the effect of *E. faecalis* infection in semen.	[26]
2018	Normospermic Oligoasthenozoos-permia	iTRAQ	Dysregulated proteins involved in metabolism, transport, antioxidation, and immune response were identified in oligoasthenozoospermia.	[27]
2017	Seminal exosomes	LC-MS/MS	Describes the seminal exosomes proteome.	[28]
2016	Adolescents with varicocoele	LC-MS/MS	Specific biomarkers of spermatogenesis and homeostasis are observed in adolescents without varicocoele.	[29]
2016	Normospermic	CID LC-MS/MS	Provides identification of large-scale N-glycosylation mapping of the glycoproteins, glycosylation sites, and glycan compositions.	[30]
2016	Smokers Non-smokers	LC-MS/MS	Smoking altered protein levels linked to inflammatory state in the accessory glands and testis.	[31]
2015	Healthy men	LC-MS/MS	Established the first large scale N-linked glycoproteome of human seminal plasma.	[32]
2015	Normospermic	LC-MS/MS	Proteome in SP indeed reflected semen oxidative stress, and mucin-5B can be a potential biomarker of oxidative stress.	[33]
2014	Fertile males Infertile males	SELDI-TOF-MS	Ten seminal proteins that are significantly upregulated in the infertile group were observed.	[34]
2013	Healthy donors Infertile men	LC-MS/MS	Identified proteins that help protect against oxidative stress and were uniquely present in the seminal plasma of the ROS-negative men.	[35]
2013	Healthy men Spinal cord injury (SCI) patients	2D/SDS-PAGE	SCI was responsible for alterations in seminal plasma protein profile leading to a deviation from homeostasis.	[36]
2013	Adolescents before and after varicocelectomy	ESI-QTOF MS/MS	19 spots were differentially expressed between pre- and post-surgery, and varicocelectomy is indeed associated with changes in protein profile.	[37]
2013	Adult men before and after varicocelectomy	LC-MS/MS	Function analysis of differentially expressed proteins demonstrated a shift back to homeostasis after varicocelectomy.	[38]
2012	Men with different spermatogenic impairment	2D DIGE	The identified proteins, especially PAP, have a strong potential to be used as azoospermia markers.	[39]

**Table 2:** Functional microRNAs presented in seminal plasma.

Symbol	Function and Significance	Reference
miR-210-3p	May serve as a sensitive and non-invasive biomarker of Sertoli cell-damage in varicocoele	[58]
miR-34b	Decreased seminal microRNA-34b as an indicator of lower semen concentration	[59]
miR-424	Down-regulated miR-424 in infertile men may induce spermatogenic cell apoptosis and sperm DNA damage	[60]
miR-371a-3p	miR-371a-3p expression level significantly correlates with sperm concentration and the total sperm count	[61]
miR-210-3p	Seminal plasma miR-210-3p is a useful clinical biomarker for dyszoospermia caused by varicocele	[62]
let-7b-5p	let-7b-5p inhibits glycolytic activities through targeting *AURKB* in asthenozoospermia	[63]
miR-192a	Seminal plasma miR-192a may serve as a potential biomarker to predict the presence of spermatozoa in nonobstructive azoospermia following varicocelectomy	[64]
miR-122-3p and miR-141-5p	miR-122-3p and miR-141-5p in seminal plasma are stable and have values for the diagnosis of asthenospermia	[65]
miR-151a-5p	miR-151a-5p was significantly increased in severe asthenozoospermia and negatively modulates mitochondrial respiratory activity, adenosine triphosphate (ATP) level and Cytochrome b (*Cytb*) mRNA and protein levels	[66]
miR-424/322	miR-424/322 is down-regulated in seminal plasma from patients with severe DNA damage	[67]

## Abbreviations

DEP	Differentially expressed proteins
LC-MS/MS	Liquid chromatography (LC)-tandem mass spectrometry (MS)
HPLC/MS	High Performance Liquid Chromatography coupled mass spectrometry
CID LC-MS/MS	Collision-induced dissociation, Liquid chromatography (LC)-tandem mass spectrometry
SELDI-TOF-MS	Surface-Enhanced Laser Desorption Ionization Time-of-Flight mass spectrometry
2D SDS-PAGE	Two-dimensional sodium dodecyl sulfate-polyacrylamide gel electrophoresis
iTRAQ	Isobaric tag for relative and absolute quantification
1D-PAGE	One dimensional (1D)-polyacrylamide gel electrophoresis (PAGE)
DEPs	Differentially expressed proteins
ESI-QTOF MS/MS	Electrospray ionization-quadrupole/time-of-flight tandem mass spectrometry
2D DIGE	Two-dimensional differential gel electrophoresis
